# Using Machine Learning for Dynamic Authentication in Telehealth: A Tutorial

**DOI:** 10.3390/s22197655

**Published:** 2022-10-09

**Authors:** Mehdi Hazratifard, Fayez Gebali, Mohammad Mamun

**Affiliations:** 1 Department of Electrical and Computer Engineering, University of Victoria, Victoria, BC V8W 2Y2, Canada; 2 National Research Council of Canada, Government of Canada, Ottawa, ON K1A 0R6, Canada

**Keywords:** telehealth, IoT security, dynamic authentication, continuous authentication, machine learning, deep learning

## Abstract

Telehealth systems have evolved into more prevalent services that can serve people in remote locations and at their homes via smart devices and 5G systems. Protecting the privacy and security of users is crucial in such online systems. Although there are many protocols to provide security through strong authentication systems, sophisticated IoT attacks are becoming more prevalent. Using machine learning to handle biometric information or physical layer features is key to addressing authentication problems for human and IoT devices, respectively. This tutorial discusses machine learning applications to propose robust authentication protocols. Since machine learning methods are trained based on hidden concepts in biometric and physical layer data, these dynamic authentication models can be more reliable than traditional methods. The main advantage of these methods is that the behavioral traits of humans and devices are tough to counterfeit. Furthermore, machine learning facilitates continuous and context-aware authentication.

## 1. Introduction

Telehealth is the distribution of health-related services and information using telecommunication technologies and Internet of Things (IoT) devices. This opportunity allows patients to have admission, advice, care, education, and remote monitoring. In this way, telehealth provides quality medical care for stay-at-home patients and remote communities [1]. The increased use of telehealth in recent years has indicated a need for a reliable and secure system. Telehealth uses online platforms to transfer and store information; however, preserving security and confidentiality is complex. In 2019, only 1% of patients used telehealth; while in the year 2020, after the COVID-19 outbreak, more than 38% of health specialists visited patients through telehealth systems [2].

The lack of investigations, implementations, and evaluations of data protection approaches in telehealth, and the flaws in cyberspaces, make it possible for intruders to gain unauthorized access to health information. The lack of consideration for data security and privacy is especially disconcerting in medical settings where confidentiality is paramount, and data corruption can prove fatal. Security and privacy often come as afterthoughts to the designers of telehealth systems; thus, many areas of input, data, and output protection are deficient, and protecting user interactions (and access to the devices themselves) is insufficient in many of the proposed systems.

In the US, the Health Insurance Portability and Accountability Act (HIPAA) [3] was developed to protect (and provide rules for accessing) the patient’s information. Authentication provides access control for systems by controlling whether a user’s credentials align with the available records on the server. Traditional security controls rely on static authentication methods [4], such as passwords, login patterns, or personal identification numbers (PINs). Besides the simplicity and accessibility of these methods, they are vulnerable to impersonation [5]. To address this issue, dynamic authentication can play a significant role in ensuring that only authorized persons or devices can connect to the telehealth system’s information and applications. Dynamic authentication uses dynamic traits that can change in each session to authenticate users and devices.

Machine learning (ML) provides a key solution to using dynamic authentication. ML can be established to lessen security traps and address security issues. Biometric traits have been used widely in many authentication platforms. Even if the human mind can realize the relationship between biometric features, ML can handle them more reliably and at-scale. ML is a significant tool for extracting concepts behind available data. Moreover, ML models can be trained to identify patterns in data and find relationships between input data and automate routine processes. This way, ML can extend the extracted knowledge in available data to make decisions or predictions on unseen cases. To protect the user’s massive private data in the telehealth system, it is extremely critical to design reliable privacy and security protection mechanisms that can accurately authenticate users. Users in smart environments include humans or IoT devices. Data collection for training models is a pillar of all ML algorithms; this technology can leverage biometrics or physical layer features to authenticate human or IoT devices, respectively [5].

The biometrics of humans involves their physiological or behavioral traits, which can be used to train an ML model for user authentication in digital healthcare systems. Physiological characteristics can be taken into account by scanning features, such as fingerprints [6], palm prints [7], or irises [8]. Furthermore, behavioral biometrics observe the user’s behavior when using the system. Most of these biometrics have already been implemented into smart devices such as smartphones. Regarding the importance of personal data in the healthcare system, we can leverage the power of ML on biometrics to increase the security of telehealth [9]. However, physiological biometrics are more at risk of counterfeiting; an advantage of using behavioral biometrics for authentication is that they are more difficult to imitate. Furthermore, collecting behavioral features in most cases does not require extra hardware and scanners that can decrease the solution costs [10].

The physical layer features in the Internet of Medical Things (IoMT) devices (mostly used for authentication) include spatial correlations of wireless channel characteristics, such as channel impulse response(s) (CIR), received signal strength indicators (RSSI), channel state information (CSI), and media access control (MAC) addresses [10]. CIR depends on the parameters and dynamic noise and fading characteristics of the communication channel [11]. CSI describes how a signal propagates from the transmitter, such as IoT devices, to the receiver in the network [12]. Therefore, CSI can make it possible to adapt transmissions to current channel conditions to achieve reliable communication in smart environments’ networks. A MAC address is a unique identifier assigned to digital devices for use as a network address in communications within a network. Furthermore, the presence of different competing MAC addresses gives information about the devices surrounding the device in question and could help in establishing context-aware authentication. A combination of the mentioned physical layer features is useful to authenticate each IoT device in the network’s communication, such as telehealth systems confidently [10].

Using biometric features in ML for authentication not only works as an added layer of protection but also allows people and devices to be identified automatically based on dynamic features in each session [13]. Another advantage of these methods is constantly controlling the system and users through continuous authentication. This way, users and IoT devices can be verified constantly to enhance cybersecurity protection on an ongoing basis. In addition, continuous authentication may provide a reinforced measure that reads users’ behavioral changes to make a secure authentication for them [14].

This tutorial investigates the applications of ML in smart environments, especially telehealth, to enhance system security. In such systems, ML-based models are applicable in making reliable authentication systems based on behavioral features or physical layer data extracted from users or physical layers. For a better understanding of ML applications in authentication, we investigated the ML life cycle and customized it to apply to authentication schemes used in telehealth. Then we categorized ML models and took some examples of recent research that employed ML to enhance this work field.

This tutorial is presented as follows: Section 2 defines the authentication concept and introduces some authentication requirements to enhance security in ever-growing environments. Section 3 investigates the ML life cycle for authentication schemes and takes some real examples of this area. Section 4 presents the hierarchy of AI, ML, and DL and categorizes the ML learning methods. We present a brief explanation of famous ML models that have primarily been used to address authentication problems. Finally, Section 5 concludes this tutorial and sheds light on further study in this area.

## 2. Authentication

Authentication is considered a key requirement for trusting the individuals and devices participating in a telehealth environment. A single compromised device can be turned malicious and bring down the whole system or cause a major loss to the patient. In such a big data analytic environment, traditional authentication schemes, such as Kerberos, are either not applicable due to resource constraints or lack providing high availability for thousands of connected devices in real-time [15]. Moreover, devices from different manufacturers with different authentication schemes may lead to a viability challenge while integrating the authentication schemes. Furthermore, in digital healthcare systems, a huge number of medical devices with different permissions and accesses to the stakeholders, such as patients and doctors, could also greatly impact the whole system’s availability.

Single-factor authentication is the least secure method that involves the user or device submitting an ID and a password. The ID could be a username, email, or device’s unique ID. As passwords or patterns are prone to be disclosed, multi-factor authentication (MFA) has gained more attention to increase the assurance of authentication for networks, services, or applications. MFA can include two or more of the following factors:

1. What you know, such as a secret password or lock pattern.
2. What you have, such as a smartphone or a smart card.
3. What you are, such as the user’s biometrics (fingerprint) or a device’s unique ID.
4. The context you are in, such as location, the activity you are doing or not doing, etc. For example, some cell phones will not allow you to use your phone if it detects you are in a moving vehicle; if you are in Canada, it should be investigated if someone wants to log in to your account from somewhere in Europe.

**Figure 1:**
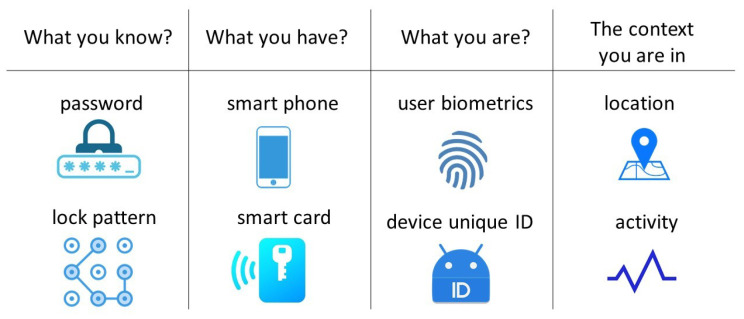
Some examples of factors that are used in multi-factor authentication.

Figure 1 provides some examples of factors that can be used in MFA techniques.

Figure 1 shows some examples of commonly used features in MFA. We are now familiar with the two-factor process of logging in to an online service, such as telehealth, where the user starts the process by attempting to log in using a username and password. The server then sends a one-time access code to the user’s smartphone and waits for the user to enter that code that remembers “what you have”. A combination of the password and the transferred code can raise the security level for authentication. In the literature, using smart devices to help authentication has been referred to as “smart authentication”. Bhunde et al. [16] developed an application for bus pass renewal and web security using smart authentication. They considered a cell phone as the second authentication factor to perform a web login on a PC. To this end, the authors proposed a smart authentication prototype that included a Java-based web server, a Chrome browser extension, and an Android application. Agrawal et al. [17] provided three security checks for mobile authentication, including matching the three-dimensional angle of the mobile, shape of the password pattern, and time taken to draw the pattern on a cell phone, which is a behavioral biometric for users.

MFA is preferred for interactions by enabling user-friendly, fast, and reliable authentication for accessing the applications [18]. “What you know” and “what you have” factors require user interaction to be authenticated in a system. “What you are” and “the context you are in” factors help to unobtrusively monitor and authenticate users throughout their interactions with mobile devices [19]. This concept has been referred to as context-aware authentication, which does not need the user’s attention to be authenticated [20]. ML plays a significant role in context-aware authentication. Furthermore, in continuous authentication based on monitoring all network entities constantly by dynamic features, using ML is inevitable [21].

### Need for Authentication in Telehealth System

Signal spoofing can seriously impact patient health by providing false information to physicians unable to make informed decisions on the treatment options. Techniques for detecting anomalies could include a mix of AI, strong authentication between physical sensors and servers, and physical layer authentication.

Any new technology comes with risks, threats, and vulnerabilities that need to be identified, evaluated, and mitigated in order to protect the user of the technology. As security is a painful necessity in many sectors, in this case, medical practitioners and patients in a hyperconnectivity setting will need to be in trust with the system at all times during a consultation or a medical intervention. More specifically, since an adversary could easily gain access to the biometric sensor data from an honest participant and try to spoof that data to gain unauthorized access to the system, modify the information or even cut access to the data, we need strong protection against those types of attacks (and others). We created an adaptive authentication approach, using machine learning techniques that allow attackers and defenders to be pitted against each other and to adapt to each other’s strategy (with the goal of automatically detecting unauthorized access), reducing the risks of data breaches, triggering alerts on abnormal behaviors or interactions, and finding faulty IoMT devices [22].

Dynamic authentication relies on authenticating users based on access to data patterns, extracted features from the network, applications used, and any other data produced dynamically in real-time [21]. ML plays a significant role in mining such data and extracting required features to accept or deny a user in the system. Furthermore, extracting biometric features from users and dynamic analysis of behavior patterns contribute to continuous authentication that can enhance cybersecurity protection and identity confirmation on an ongoing basis [14]. Such an authentication scheme is so comfortable for users because they only need to fulfill their regular routines in order to be identified. Since continuously tracing users is not affordable for humans, the only solution to perform ongoing authentication is by using ML. The next section provides readers with an overall insight into ML models and their applications in authentication schemes.

**Figure 2:**
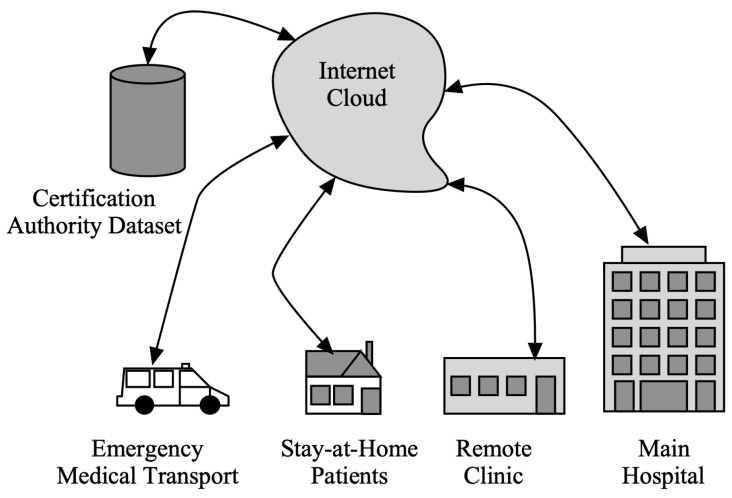
Schematic view of a telehealth system and the relationship between entities that should be authenticated.

The cyber risk assessment system for a bio-cyber-physical system (BCPS) (a.k.a. telehealth) is different from the traditional cyber-physical system (CPS). In a telehealth environment, where humans (e.g., doctors) and CPS (e.g., Internet of Medical Things (IoMT) devices) work together to accomplish a task (e.g., operating on a patient), the nature of the relationship can be addressed as an interaction between two systems: the human (biological) and medical devices (cyber-physical) [23]. Physically unclonable functions (PUFs) is the preferred option for endowing hardware components with unique identities [24]. Figure 2 demonstrates the available entities in a telehealth system and the overall schema that can be considered for authentication.

A telehealth system could potentially consist of the following components, from left to right:

1. Emergency medical transport where paramedics need to alert the main healthcare facility and perhaps share the patient’s vitals with emergency room personnel to better deal with the emergency and prepare the needed resources at the hospital.
2. Stay-at-home patients where elderly patients could recover better at their homes and to reduce healthcare delivery expenses and hospital beds.
3. Remote clinics with general practitioners and nurses desire to provide quality healthcare at remote distant communities or disaster recovery areas.
4. Main hospital or central healthcare delivery system.

It is vital that several security features must be provided to all these components to ensure delivery of quality healthcare and thwart potential attacks, miscommunications, or wrong patient identity.

## 3. Machine Learning Life Cycle for Authentication

**Figure 3:**
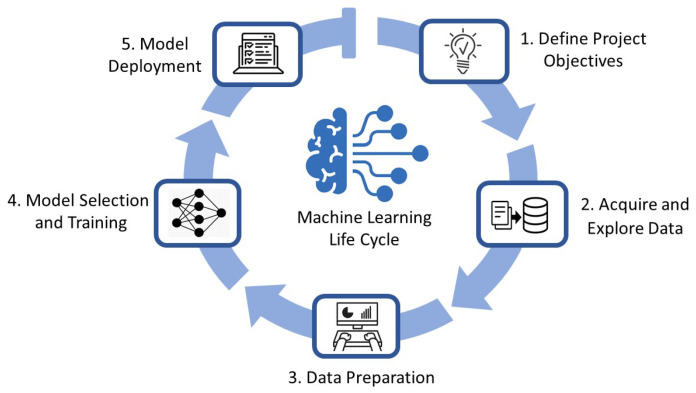
Machine Learning Life cycle Schematic View.

The ML life cycle is the cyclical process of deriving the practical value of using ML in defined criteria. It can perform end-to-end processing and give a perspective of how an entire project should be structured to approach reliable results in practical problems. Figure 3 depicts a schematic view of the ML life cycle in five high-level stages. In this section, we tailor this roadmap to address authentication problems by ML and elaborate by taking some pragmatic examples.

### 3.1. Define the Project Objectives

The first step in addressing a problem is scoping and selecting the relevant use cases to define the project objectives. To design a reliable authentication platform in telehealth, we need to define the objectives and constraints for data analysis and modeling that can discriminate a user. It is imperative to investigate the required authentication scenarios. In a telehealth environment, where doctors and IoMT devices work together to operate on patients, the nature of the relationship can be addressed as an interaction between human and medical devices, respectively, with biological and cyber-physical traits [23]. For example, each IoMT device must possess a unique ID that is anti-tamper resistant. Such devices should be authenticated using MFA. Using a PUF for the device ID also helps in establishing secure session key exchange. Using physical layer data from IoT devices in the network to continuously authenticate each node in the network of a telehealth system needs planning to collect data from IoMT and connected devices, such as patients visiting platforms. Furthermore, we need to collect data that can show the device owner’s personal behavior and define an ML model that can discriminate users based on the gathered data. Defining project objectives are not confined to data collection and encompass all stages until model deployment. Using smart planning can increase the probability of a project’s success and decrease the project’s costs.

### 3.2. Acquire and Explore Data

Data effectiveness is a pillar to training an ML model that operates efficiently for human and device authentication in telehealth. Therefore, extracting discriminative features that can be considered as each person or device’s signature for authentication is essential. In the first stage, the required data for authentication are collected by an application or scanner on the users’ devices. In the next stage, specific behavioral features are extracted from the data collected. Siddiqui et al. [5] compared many behavioral-based biometric systems to conclude which features work best for authentication. Following, we will investigate some simple data that can be used to authenticate people or IoT devices.

Keystroke dynamics are typing patterns useful for authentication of the system users [25]. The touchscreens of mobile devices allow collecting features ranging from the finger area to screen pressure, or time-based features. Mouse movement is another type of biometric-based authentication information useful for the continuous monitoring process and authentication on desktop devices [26]. Typically, clicking actions, timing, and the movement direction of the cursor can constitute a user profile for authentication. Since granting access to the secure information of patients to doctors, nurses, and other parties in the telehealth platform is a critical task, it is noteworthy to monitor them continuously from their typing and clicking behavior to detect and prevent anomalous activities in the system.

The physical activity and movement of a person is another reliable behavioral biometric that can identify them using ML models [27]. Accelerometer, gyroscope, and magnetometer are three embedded sensors available in most smartphones often used to recognize the activity of smartphone users, such as running, walking, sitting, lying down, etc. [28], so-called human activity recognition (HAR). This data can also be applicable for authenticating smartphone users with high confidence [27]. Furthermore, other sources of HAR data can be used to train ML models for both behavioral traits analysis [29] and user authentication. These data can also be collected from wearable sensors [30], such as smartwatches and camera devices, such as Kinect [31,32]. Moreover security surveillance [33] and authentication [27], the mentioned HAR data can be useful for healthcare systems [30], smart environments [33], remote care to elderly people living alone for smart healthcare [33], etc.

In addition to the mentioned behavioral traits, many human physiological features can be used to authenticate a person. Human fingerprints [6], the face [34], eye movement [35], ECG heart signals [36], and electroencephalogram (EEG) brain signals are examples of the most commonly used features in smart environments and mobile devices for authentication. Most smartphones are equipped with a fingerprint scanner and front camera to capture face images or iris patterns for authentication. In some devices, such as virtual reality (VR) headsets, eye movement features are extracted to authenticate the device’s legitimate owner for access to bank accounts and in-app purchases [35]. Moreover, through ECG authentication in smartphones, users only need to touch two ECG electrodes (lead I) of the mobile device to be authenticated by the ML algorithm [36]. Collecting EEG signals are more complicated as EEG electrodes need to be placed on the human scalp. On the other hand, for the sake of mental conditions, unique EEG features are very robust and secure to be used in the authentication process by an ML model [37].

### 3.3. Data Preparation

Depending on the data and the ML method for authentication, some preprocessing, such as feature selection, feature extraction, data integration, and data cleaning, are needed to enhance authentication performance. Performing feature selection and extraction can help deal with high-dimensional data and avoid overfitting in model training [38]. Handling incomplete data, missing values, outliers [39], and anomalous samples [40] are other practical preprocessing techniques for data cleaning. Annotating samples is also required to use supervised models to authenticate persons and devices.

Authentication datasets are very prone to imbalance conditions. The most conventional classifiers and authentication schemes assume equally balanced classes [41]. Since the imbalanced dataset might lead to poor performance after training the model, which is so important in telehealth authentication, it is useful to check for enough instances from each class or use other methods, such as resampling, to handle imbalance conditions [42]. Moreover, employing metrics that can handle the imbalance condition can be useful for model training until approaching a stable model with available data. Such metrics will be discussed in the next section.

Inspired by the AdaBoost algorithm, Tran et al. [41] proposed an approach to handling the class imbalance issue in biometric authentication systems. First, they trained weak one-class classifiers using data from both classes. They then combined weak classifiers to improve the overall classifier performance without causing overfitting. Instead of using different datasets to make diversity in the weak classifiers, they used different parameters in their algorithm. Kim et al. [43] proposed a hierarchical classification model to mitigate the issue of imbalanced class sizes in biometric data, specifically in healthcare. They managed the issue of imbalanced class sizes in the biometric dataset by reorganizing the classes into a hierarchical structure and designing a deep learning-based classifier. Lu et al. [44] proposed a privacy-preserving federated learning framework to improve the diagnostic accuracy of decentralized machines for biometric authentication in imbalanced class conditions without data transfer.

### 3.4. Model Selection and Training

To address dynamic authentication in telehealth, we face a complex problem involving a plethora of data and lots of variables. Although ML would still be the best approach, choosing the best model to deal with available data is a significant task. We must step through data analysis and ML workflow to choose the best model for addressing the problem at hand. For example, keystroke dynamics data points contain separate features that can be used in most ML models, such as SVM, NB, RF, KNN, and regular ANN. However, to analyze ECG signals, it is better to use models that can handle sequential data or time series, such as the hidden Markov model (HMM), long short-term memory (LSTM), and the convolutional neural network (CNN). In some authentication schemes containing stationary data, such as face recognition, the best choice can be to use CNN. While in traditional ML methods, it was prevalent to extract some features from images and then proceed with other ML methods. For example, Fard et al. [45] employed an autoencoder to reach the best feature space for discriminating each user in the system and authenticate them based on their locally linear reconstruction error. Since their proposed method is very low cost, it can be a good choice to be used in telehealth authentication systems where we need to authenticate users in real-time.

**Figure 4:**
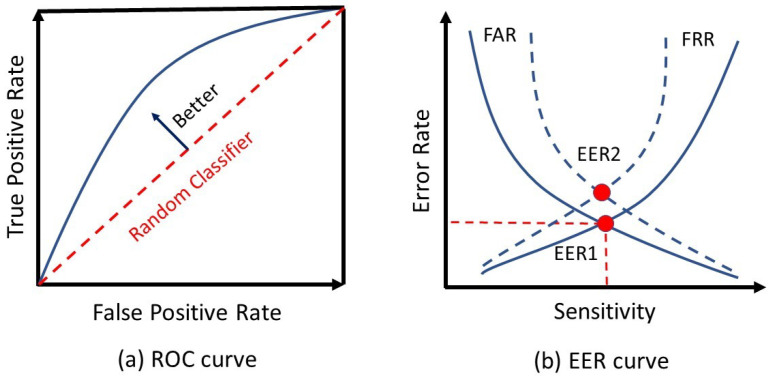
(**a**) ROC curve based on false positive and true positive rate, (**b**) comparing EER for two biometric systems.

Moreover, the model’s result quality is a fundamental factor that must take into account to select a model. Regarding the problem, different metrics could be useful for evaluation. Despite the popularity of the “accuracy” metric, it is not appropriate when working with imbalanced data that the number of samples is much different in classes, such as authentication problems. The area under the ROC curve, precision, and recall are popular metrics for model evaluation in imbalance conditions, such as authentication problems. Figure 4a depicts the ROC curve (the greater the area under this curve, the better the final model). The ideal condition is when it reaches the upper bound with the value of one. Moreover, the assessment of a biometric model is determined through three parameters of false acceptance rate (FAR), false reject rate (FRR), and equal error rate (EER). FAR and FRR show the percentages of false users authorized and the percentage of legitimate users rejected by the model, respectively. While EER refers to the threshold values for FAR and FRR and shows the point at which the FAR is equal to the FRR. A lower EER indicates a more accurate biometric system. Figure 4b depicts the EER for two biometric systems [46]. In this diagram, the system represented with the solid lines shows better results than that represented by dashed lines.

The training procedure can be offline in authentication schemes and having a long-time learning model is not a big challenge. On the other hand, in such problems, the inference time is critical, and the trained model should accept or reject the user in the system in real-time. For example, k-NN is a lazy model and does not contain any training phase, while each time it wants to evaluate a user, it should calculate the distances and define the neighbors, it is not proper to address a real-world authentication scheme in telehealth. Contrarily, training deep models take time, but their response time is reasonable, and they are good choices for an online authentication scheme. From another point of view, we can consider the dimensionality and the number of available samples for training a model. For example, SVM is suitable for problems with high dimensionality, but it cannot handle many samples. In contrast, deep models need much more samples for training, and the sample dimensionality depends on the model’s structure.

### 3.5. Model Deployment

Finally, we approach an authentication scheme that can respond in real-time and performs well on evaluation metrics. In smart environments, such as telehealth systems, it is more applicable to implement an authentication system that can evaluate users continuously without wasting time. As a result, the deployed model should be able to collect required data, process, and extract features automatically, and make a reliable decision based on the system requirements. For example, to continuously authenticate doctors, the system can consider their voice while talking, their keystroke dynamics while writing a prescription, and their faces while visiting the patients. A combination of such features also can elevate the system’s security. As well, the physical layer data can be extracted to authenticate the smart devices available in the network.

Testing the deployed model and monitoring its performance, respecting the evaluation metrics and inference speed to ensure that it works as expected on new data, are necessary to have a reliable authentication system. Moreover, having a maintenance plan to solve the probable issues and system flaws can raise system confidence.

After deployment, it is necessary to keep a product up to date. Most ML-based authentication schemes need to train the model using data from all available distributions. On the other hand, the trained model cannot perform well on data from a new distribution. As a result, enrolling a new user in the existing trained authentication model is a crucial task. Ivanciu et al. [47] proposed an ECG-based authentication system using Siamese neural networks to address this problem. In such systems, the model is trained based on many couples of samples from “the same class” and “different classes”. So, the main structure comprises a twin network and a binary classifier that returns “positive,” which means two inputs are from the same user, and “negative,” which indicates a big difference between them [48]. Siamese networks are not sensitive to adding new users to the system because they consider the similarity between the input couples for decision and do not need to see all classes while training. Since the authentication system needs to store at least a sample from each legitimate user in a repository for the next comparison, in case of changing the biometric features of a person during the time (concept drift), such as aging, it is required to update the database. In this case, the Siamese network is more robust and does not need to renew the model training, while other ML methods need to keep updated.

## 4. Machine Learning Models in Authentication Schemes of Telehealth

ML is a subfield of artificial intelligence (AI) that learns from available data through the training phase [49]. As a result, algorithms can learn without explicitly being programmed. Data are the pillars of training reliable ML models. Therefore, to develop an ML-based authentication model, it is important to use features that represent users and can be considered their signature. This way, the ML model can learn how to discriminate each user from others or compare their new samples with the previous one to authenticate them. In most cases, it is needed to perform some preprocessing, such as data cleaning, denoising, outlier detection, handling missed values in collected data, and feature selection and extraction.

**Figure 5:**
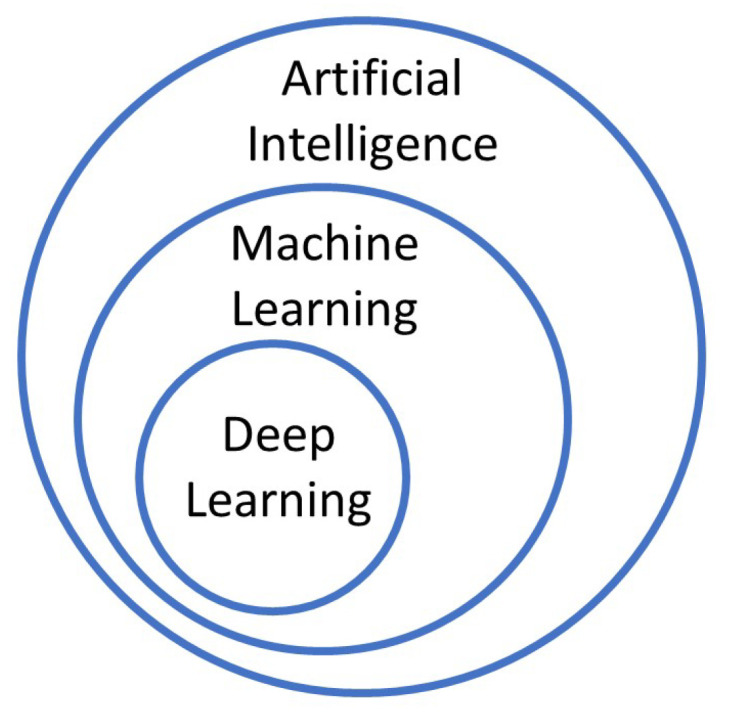
Hierarchy of artificial intelligence, machine learning, and deep learning.

Emerging deep learning (DL) in the recent decade is evolutionary in ML models. The main advantage of DL is the power to extract relevant features related to the problem at hand. DL is flexible, adaptive, and can extract features to achieve excellent performance. Figure 5 shows the general hierarchy of the three main concepts of AI, ML, and DL [50]. We need this concept to define the role of algorithms in authentication problems. AI covers a vast category of models that may be built based on an expert’s knowledge or learned to extract concepts from available data. ML contains part of these models that may be trained based on data to analyze data or predict future events in unseen data. DL includes a branch of ML models based on artificial neural networks (ANNs) containing some layers to extract features automatically, and decide based on them.

### 4.1. Machine Learning Categorization

In the following, we will introduce ML categories and investigate some famous models in each category that has been used widely in state-of-the-art to address authentication problems. This categorization provides insight into the roles of different ML models in the authentication. Since these authentication techniques use dynamic data from human or IoMT devices, they can be used widely in telehealth.

#### 4.1.1. Supervised Learning

**Figure 6:**
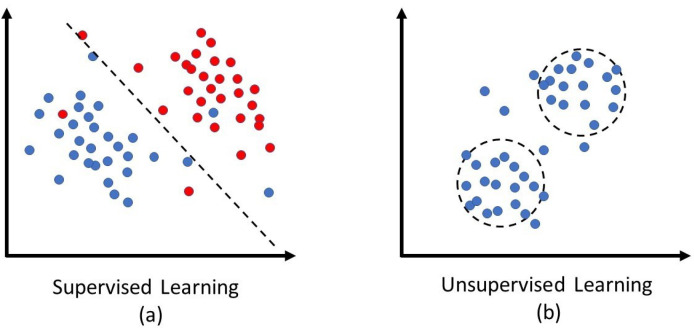
Schematic view of classification vs. clustering. (**a**) Blue: class-1, red: class-2, supervised models learn patterns to discriminate data samples based on their labels. (**b**) Unsupervised model cluster samples (just based on their similarities).

In supervised learning, a model learns an inferred function from annotated samples to predict output values or forecast future events. In the training phase, the output of the model is compared with the correct targets to find errors and modify the model accordingly. The final model can generalize predictions for new inputs. Figure 6a shows a simple view of supervised learning just to imagine learning from the annotated samples [51].

In supervised authentication, an ML model can use data from an intended person and other individuals for training. Assume users’ typing patterns are monitored. Keystroke dynamics [25] are representative patterns for each user. In model training, the keystroke dynamics features are considered the model input, and the identity of each person is the model output. The model learns how to map each person’s specifications to his/her identity. Moreover, the trained algorithm can define whether the person is genuine or an imposter. In telehealth, accessing patients’ data is a critical action that this authentication technique can handle. Therefore, the people who want to access critical data are monitored continuously from their dynamic typing patterns to give permission, and anytime their typing patterns do not match with the registered samples, the permission will be suspended, and a higher level of security will be needed to continue their activity.

Naïve Bayes (NB) [52], k-Nearest Neighbor (k-NN) [52], random forest (RF) [53], and support vector machine (SVM) [52] are popular supervised ML methods that have been widely used in the literature to address authentication problems [4].

NB is a simplified probabilistic ML method for classification tasks. The features of the problem were assumed to be independent and equal for simplification, and the presence of a feature cannot affect the frequency of other features. In authentication problems, NB determines the probability that a user is genuine, given the known probabilities extracted from training data in the model. Estrela et al. [54] proposed a framework using touch dynamics biometrics for continuous authentication in mobile banking applications. Touch dynamic is a biological recognition method based on individuals’ touch patterns. In that research, NB outperformed other methods among six ML models including RF, SVM, gradient boost (GB) [55], Extreme Gradient Boosting (XGB) [56], Naive Bayes Bernoulli (NBB) [52], and Naive Bayes Gaussian (NBG) [52].

k-NN is a lazy and non-parametric classifier that uses proximity to define k nearest neighbors of an individual data point. Lazy models in ML defer data processing until receiving a request to label a new example [52]. Then they will annotate the new example based on the majority labels of instances in the intended example neighborhood. Moreover, non-parametric ML models do not make strong assumptions about forming a mapping function and are free to learn any functional form from training samples. Wang et al. [57] used k-NN to authenticate individuals through touch dynamics. We need to have previous instances from each person in the database to authenticate them. When a new data point is entered, for example, if k is five, the five closest data points to the new point are chosen, and the majority of the data point labels can annotate the new instance.

RF, as an ensemble ML method, has been used widely in many authentication schemes. Smartphone user identification [58], continuous authentication in mobile devices [59], using smartphone sensors and keystroke dynamics for authentication [60], bimodal behavioral biometric authentication [61], touch dynamic authentication [62], and multimodal smartphone user authentication [63] are some new research methods used in RF to authenticate users. Belgacem et al. used RF for human authentication with electrocardiogram (ECG) data (used widely in healthcare). RF is an ensemble of multiple decision trees that are slightly different from each other, considering sample sets used to train each model. Since bagging is used for sub-sampling, RF can ensure that the behavior of each decision tree is not (too) correlated with other decision trees. To compare the strengths of ML algorithms, Almalki et al. [64] analyzed mouse click streams for online continuous authentication using NB [52], k-NN [52], and RF [53], in which RF outperformed other models.

SVM is a robust supervised classifier that has been used in many authentication schema, such as touch dynamic [65], and swipe gesture authentication [66,67]. Ismail et al. employed SVM to authenticate patients, especially elders, in the smart healthcare system using their voice signal [68]. SVM aims to find a hyperplane in the feature space that distinctly classifies data points with maximum margin among many possible hyperplanes. Maximizing the margin distance provides more confidence for the classification of unseen data points. In binary classification tasks, the data points can be labeled as genuine or imposter, and maximizing the distance between these two groups can guarantee the model’s generalization. Specifically, SVM is more effective in high-dimensional spaces and can be used with many Kernels. Hinge loss is the loss function used in SVM to maximize the margins between classes.

#### 4.1.2. Unsupervised Learning

Unsupervised learning aims to cluster and analyze unlabeled data. Unsupervised models can discover hidden patterns in data without human intervention and any label as guidance. Their ability to explore differences and similarities in information makes them a potential solution for anomaly detection. Figure 6b shows a simple view of unsupervised learning for clustering just to imagine training a model based on the similarity of samples. As a result, imposter data points are not confined to clusters’ boundaries and can be defined as anomalous or outlier data samples. In recent years, gait data have been used widely for authentication purposes [69,70]. For example, Cola et al. [71] used the user’s gait pattern automatically when a device owner starts wearing that in a healthcare system. To authenticate the device owner, any gait behavior far from the learned pattern is considered an anomaly. Tan et al. [72] proposed an unsupervised anomaly detection scheme for authentication, and so on; they deployed a certificateless authentication technique for conditional privacy-preserving. Gebhardt et al. [73] also employed unsupervised anomaly detection for document authentication. Chen et al. [74] used clustering in physical layer data from edge computing systems for authentication IoT devices that can be very effective to detect intruder IoMT devices in telehealth.

Dimensionality reduction techniques, such as principal component analysis (PCA) and singular value decomposition (SVD), are also unsupervised methods used for feature extraction. The results of all methods discussed above are highly relevant to the set of input features of the model. As a result, PCA and SVD can help find the proper feature space to search for the best hypothesis to solve the problem. On top of all mentioned methods, deep learning models are able to extract the best feature set related to the problem’s goal. Nakanishi et al. [75] tackled the effects of PCA feature extraction in brain waves as unconscious biometrics used for continuous authentication. Moreover, Muratyan et al. [76] used PCA for feature extraction to propose a multi-modal user authentication system in IOT wearables for health-tracking. SVD is mostly applicable in image-based feature extraction. For example, Yu et al. [77] proposed an SVD-based authentication scheme. They used SVD to decompose image data into three matrices of left singular value, right singular value, and singular value and performed authentication through a value calculation method based on the singular value matrix.

#### 4.1.3. Semi-Supervised Learning

Semi-supervised learning offers a method between supervised and unsupervised learning. Semi-supervised learning uses a smaller annotated dataset while training to guide classification and continues with a larger unlabeled dataset to fine-tune the model. In some problems, such as authentication, there is no access to enough labeled data to train a supervised model; therefore, semi-supervised algorithms can address this issue. Yildirim et al. [78] used a semi-supervised method of learning from behavioral biometrics of mouse dynamics data for authentication. This behavioral authentication model is very applicable to authenticate legal operators of telehealth platforms while connecting with patients or accessing their data. Moreover, Kaiafas et al. [79] employed a semi-supervised outlier detection method for authentication.

#### 4.1.4. Reinforcement Learning

Reinforcement learning (RL) algorithms learn from trial-and-error search and delayed rewards. RL models interact with the environment to exercise rewards and penalties and automatically determine the ideal behavior to maximize the defined performance metric. This reward feedback in the RL model can be used to find the best action for classification [80,81,82], feature selection [38], or any other required decision in the system. Cui et al. [80] used RL to propose an adaptive authentication scheme. They developed a multi-factor authentication method that uses different combinations of authentication models proportionate to the level of authentication confidence requirements. Xiao et al. [81] used RL for authentication in controller area networks (CANs) in smart environments using physical-layer data. Moreover, Xu et al. [82] used voltage data in a similar RL platform for the authentication process in CANs. This trend can be very useful to trace the activity of legal IoMT and connection devices in telehealth platform.

### 4.2. Deep Learning (DL)

**Figure 7:**
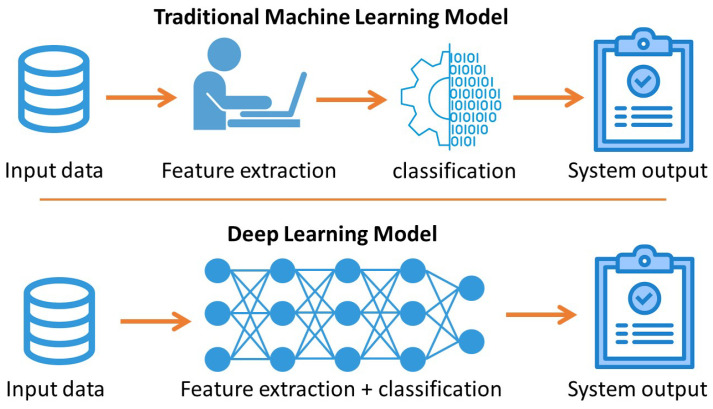
A comparison of deep learning and traditional machine learning models.

The main advantage of DL is the ability to extract features regarding the problem’s requirements, which is a challenge in other ML models. Figure 7 depicts the overall views of traditional ML models and compares them with a DL model [83].

A deep neural network (DNN) extends the number of hidden layers in an artificial neural network (ANN) to empower feature extraction ability which is the main advantage of DL over other ML methods. Using multiple layers in DNN can help extraction of higher-level features from the raw input progressively. For example, dense networks fit the problems containing independent and identically distributed (IID) data points despite time series and stationary data points. Moreover, dense layers are mostly used in the network’s final stages to conclude the entire network’s result. CNN is another famous architecture effective for analyzing data with stationary specifications such as visual imagery [84]. CNNs are regularized versions of dense networks which are confined to the number of parameters in each layer by tying them to each other. As a result, it is enough to train a fixed-size filter’s parameters instead of learning the weights of all connections. Facial images, fingerprints, and sequential multi-dimension data from accelerometers are examples of data to train models based on CNN structures. For instance, three-dimensional data from accelerometer, gyroscope, and magnetometer sensors may constitute a nine by *n* matrix that can be processed in a CNN-based model [85]. Such data is very effective in both gait recognition of patients for disease diagnosis or using in dynamic authentication system at the same time for patients and doctors in the telehealth system.

A recurrent neural network (RNN) is an architecture to handle temporal dynamic behavior with feedback connections. RNN is mostly used in time series analysis having variable length sequences of inputs [86], such as NLP problems. LSTM is an improvement in RNN designed to remember or forget values over arbitrary time intervals. As a result, LSTMs are insensitive to time series length that can process data sequentially and keep its hidden state through time. As an example, LSTM is a useful structure for analyzing time series, such as ECG and EEG signals for disease diagnosis and authentication in a healthcare platform.

The mentioned structures are only three models widely used in many fields, such as authentication. In a DNN, either of these structures or a hybrid can be used, while the learning procedure is the same as the previously discussed methods. Deep models contain similar categorizations to ML, which are discussed in the following.

#### 4.2.1. Supervised DNN

As mentioned, a supervised model is trained based on input features and available labels. As well supervised DNN models follow the same concept (regarding the data type), they can use either neural network structure. As a result, annotated sample dense network classifiers suit episodic data points. CNN classifiers are best for stationary data and LSTM classifiers fit the time series and inertial signals where input data sequences depend on the adjacent values. In the literature, several hybrid models have also been proposed for authentication purposes [69,70].

Zeroual et al. [87] used a deep CNN classifier to authenticate people based on their face images. To handle complex computation in this model, they allocated the training process to the cloud because of the huge amount of data. Abuhamad et al. [88] investigated LSTM classifiers in three different architectures of simple LSTM, bidirectional LSTM, and multi-layers LSTM for user authentication. The dataset was collected from participants using readings of accelerometer, magnetometer, and gyroscope sensors from cellphones with a high authentication frequency. Both mentioned supervised models used dense networks in their last layers for classification. Xia et al. [69] combined LSTM and CNN layers to recognize human actions in mobile and wearable devices. In their architecture, the raw data from the accelerometer and gyroscope was fed into an LSTM followed by CNN layers to make a robust classifier. Since IoMT wearable devices are prevalent in digital healthcare systems, all of the mentioned models are effective in such use cases.

#### 4.2.2. Unsupervised DNN

Autoencoder is one of the most common unsupervised models in DNN. In this architecture, a bottleneck is imposed in the middle of the network to force a compressed knowledge representation of the input, i.e., encoded data. Autoencoder takes an unlabeled dataset and try to reconstruct the input from encoded data. For authentication applications, the intended user is considered the genuine class, and the unauthorized users constitute impostor classes. As a result, the autoencoder is trained only with genuine data from an authorized user until the reconstruction error converges to a low value. Then, the model can detect imposter instances as their reconstruction error is higher than a defined threshold. The model can be adaptive considering the problem sensitivity by adjusting the threshold. For example, in secure environments, by choosing lower thresholds, any deviation from legal instances is detected as an intruder to the system.

Oza et al. [19] proposed a one-class classifier autoencoder for active authentication using the mentioned unsupervised technique. Since they used face-active authentication datasets, the one-class classifier was a CNN autoencoder to learn meaningful feature representations. An advantage of such networks is that they can use any pre-trained model instead of initializing network weights from scratch. This way, they can take advantage of transfer learning in DNN to expand the trained model’s ability based on similar data to the present dataset [89]. Ashraf et al. [90] designed an algorithm to recognize intrusions from the central network gateways of the Internet of Vehicles (IoVs). The proposed algorithm used data from the UNSW-NB15 dataset for external network communications and the car hacking dataset for in-vehicle communications, which is applicable for smart healthcare environments and smart tracking ambulances in such systems. As the data can be converted to sequential data, they employed an LSTM autoencoder to detect abnormal activities in the network, i.e., continuous authentication.

Giorgi et al. [91] used a hybrid model including a combination of supervised and unsupervised LSTM models for authentication. The authors used gait data analysis from cell phone sensors to perform continuous authentication. Gait analysis is mostly used to evaluate people’s dynamic posture and coordination during movement. To this end, Giorgi et al. [91] conducted some preprocessing for noise reduction, normalization, and creating a fixed sample size matrix to train the proposed model. Their hybrid model is a combination of a supervised LSTM binary classifier and an unsupervised LSTM autoencoder.

#### 4.2.3. Semi-Supervised DNN

As discussed, it is often expensive to label large datasets. Specifically, collecting real data and performing required preprocessing and annotations are very costly. However, semi-supervised models can address this problem. For example, Wang et al. [92] proposed a semi-supervised hybrid deep model to perform physical-layer authentication and detect spoofing attacks by controlling channel state information (CSI), effective to detect legal IoMT devices in the healthcare platform. They first used a CNN to extract the local features and employed an RNN to capture the dependencies between different frequencies in CSI. Then, they proposed a semi-supervised hybrid CNN and RNN deep model to extract contextual and local information in CSI for user authentication, where only a tiny part of the channel observations is annotated.

#### 4.2.4. Deep Reinforcement Learning

Deep reinforcement learning (DRL) combines the advantages of deep learning and reinforcement learning to overcome the problem of a computational agent. Wang et al. [93] employed DRL with CNN structure through facial feature extraction, transformation, and comparison for face authentication under the situation of vague facial features in mobile payment. Moreover, Shahbazi et al. [94] used DRL in the context of blockchain to authenticate IoT devices in smart environment gateway, such as smart healthcare.

Table 1 concludes this section by categorizing the applications of ML models in the authentication. In this tutorial, we just used a few examples from each category of ML models that have been used in addressing authentication problems.

## 5. Conclusions and Future Work

The development of wireless communication technologies has become important in the modern medical system, such as telehealth. In such systems, the patient’s data can be collected and sent to health professionals to obtain the patient’s status anytime and anywhere. Unauthorized access to such data may compromise the patient’s privacy, and any change in data could affect the therapy procedure. Furthermore, the presence of unauthorized devices in such a network can put the system at risk of data leakage and eavesdropping.

Adaptive biometric authentication is currently deployed in online banking, e-commerce, and payments, where frictionless real-time authentication is a must. It is not an alternative to the typical password-based authentication, but it does prevent a single point-of-security failure relying on the password and leverages continuous identity assurance requirements for any critical infrastructure. Authentication can be considered a complementary level of security enhancement to address unauthorized access issues proactively. Therefore, it is not an alternative to traditional cryptography models. Traditional adaptive authentication is rule-based, where the access and authentication adapt to the context (e.g., who, when, where) of each request, for instance, access can only be granted from a corporate, managed group. However, future adaptive authentication systems would use machine learning, advanced analytics along with rule-based authentication to cover many possible scenarios. For example, unsupervised learning can be used to create profiles for the stakeholders, e.g., doctors, by which the decision of real-time access controls will take place.

The growing scope and complexity of modern IoMT identity and access management (IAM) environments encourage the ubiquitous use of analytics. One of the most pronounced next-generation access services is adaptive authentication. However, instead of only using traditional rule-based adaptive authentication, researchers are introducing machine learning and advanced analytics to cover all the possible scenarios. For example, unsupervised learning can be used to create users’ profiles by which the decision of real-time access controls will take place. An organization can establish a baseline for a patient or a group of similar patients’ medical data, analyze the sudden changes in the patient’s data (anomaly) that behave differently, and take necessary actions.

This tutorial investigates the available machine learning methods and the categorization of the literature. The main contribution of this tutorial involved bringing machine learning models to the concept of authentication in the telehealth network and investigating their ability to handle different biometric data from human and physical layers for smart devices. To enhance the authentication scheme performance, we needed to consider the problem at hand in an entire machine learning life cycle. For this purpose, we discussed the authentication problem from defining objectives to data acquiring, data preprocessing, model building, and final deployment. In each stage, we provided examples of state-of-the-art methods that used the introduced techniques in their authentication schemes and customized the machine learning concepts for this realm. Since we have implemented many machine learning-based authentication models, for a better understanding of this topic, it is suggested that the following work sketch the prominent authentication models practically and dissect them in detail.

## Figures and Tables

**Table 1:** Categorization of the traditional ML and deep ML models and examples for further study.

	Supervised Learning	Unsupervised Learning	Semi-Supervised Learning	Reinforcement Learning
**Traditional ML Models**	NB [4,54]	Clustering [71,72,73,74]	Behavioral Biometrics [78]	Classification [80,81,82]
	k-NN [4,57]	PCA [75,76]	Outlier Detection [79]	Feature Selection [38]
	RF [4,58,59,60,61,62,63,64]	SVD [77]	—	Adaptive Authentication [80]
	SVM [4,65,66,67,68]	—	—	CAN [81,82]
**Deep Models**	CNN Classifier [84,85,87,88]	CNN Autoencoder [19]	CNN Semi-supervised LSTM [91]	CNN DRL [93]
	LSTM [69,70,86]	LSTM Autoencoder [90]	Hybrid Semi-supervised [92]	Blockchain-based DRL [94]
	Hybrid Models [69,70]	Hybrid Autoencoder [91]	—	—

## Data Availability

Not applicable.
